# Applying Lincoff’s Rule to Central Serous Chorioretinopathy: The Macula Society International CSCR Research Network (MICRoN) Report-15

**DOI:** 10.1016/j.xops.2026.101197

**Published:** 2026-04-17

**Authors:** Niroj Kumar Sahoo, Giulia Gregori, Nasiq Hasan, Sarah Abou Daher, Supriya Arora, Vishal Govindhari, Marco Lupidi, Jessica Cao, Stanley Saju, Charles C. Wykoff, Lorenzo Ferro Desideri, Marion R. Munk, Yusuf Ashfaq, Zachary Kroeger, Lihteh Wu, Jay Chhablani, Macula Society International CSCR Research Network (MICRoN), Nasiq Hasan, Arman Zarnegar, Carmen Antia, Yusuf Ashfaq, Luis Aria Barquet, Elodie Bousquet, Jessica Cao, Lisa Checchin, Peranut Chotcomwongse, Andrea Corletti, Lorenzo Ferro Desideri, Adrain T. Fung, Priyank Gandhi, Sunir Garg, Manjot Gill, Giulia Gregori, Felicia Hertkorn, Naoya Imanaga, Ninan Jacob, Samer Khateb, Rahul N. Khurana, Min Kim, Hideki Koizumi, Zachary Kroeger, Timothy Lai, Luiz H. Lima, Marco Lupidi, Bita Momenaei, Marion R. Munk, Roselind Ni, Maurizio Battaglia Parodi, Gabriele Piccoli, Lorenzo Pili, Francisco Rodriguez, Elizabeth Rossin, Paisan Ruamviboonsuk, Niroj Kumar Sahoo, Stanley Saju, Priya Shah, Rufino Silva, Panisa Singhanetr, Kent Small, Lucia Sobrin, Carol Villafeurte, Stela Vujosevic, Jay Wang, Halit Winter, Lihteh Wu, Charles C. Wykoff, Glenn Yiu, Arman Zarnegar, Micheal Zhang, Jay Chhablani

**Affiliations:** 1 Department of Ophthalmology, University of Pittsburgh Medical Center, Pittsburgh, Pennsylvania; 2 Department of Ophthalmology, Eye Clinic, Polytechnic University of Marche, Ancona, Italy; 3 Department of Ophthalmology, American University of Beirut Medical Center, Beirut, Lebanon; 4 Bahamas Vision Center and Princess Margaret Hospital, Nassau, New Providence, Bahamas; 5 Pushpagiri Vitreoretina Institute, Secunderabad, India; 6 Retina Consultants of Texas, Houston, Texas; 7 Inselspital, University Hospital Bern, Bern, Switzerland; 8 Augenarzt Praxisgemeinschaft Gutblick, Pfäffikon, Switzerland; 9 Department of Ophthalmology, Northwestern University Feinberg School of Medicine, Chicago, Illinois; 10 Casey Eye Institute at Oregon Health and Science University, Portland, Oregon; 11 Asociados de Macula Vitreo y Retina de Costa Rica, San José, Costa Rica; 12 University of Pittsburgh School of Medicine, Pittsburgh, Pennsylvania

**Keywords:** CSCR, Central serous chorioretinopathy, Lincoff’s rule, Ocular vectors, Curvature

## Abstract

**Purpose:**

To characterize subretinal fluid (SRF) accumulation patterns in central serous chorioretinopathy (CSCR) and develop a nonangiographic approach for predicting leak location, applying principles similar to Lincoff’s rules for rhegmatogenous retinal detachment.

**Design:**

Retrospective, observational.

**Participants:**

The study examined 107 eyes of 106 patients with single-leak CSCR confirmed by fundus fluorescein angiography.

**Methods:**

En face OCT images were analyzed to map SRF boundaries and configurations. A best-fit circle placement method corresponding to the presumed “primary pool” was applied to predict leak coordinates. Prediction accuracy was evaluated by comparing predicted versus actual leak locations using mean absolute error (MAE) and intraclass correlation coefficients (ICCs). The relationship between SRF morphology, leak position, and ocular vectors was investigated.

**Main Outcome Measures:**

Accuracy of the predicted leak coordinates, compared with the true leak coordinates.

**Results:**

Four distinct SRF patterns were observed: extramacular collection (4.7%), foveal extension (2.8%), macular pooling (62.6%), and inferior gravitational extension (29.9%), which could represent a hypothetical sequence of SRF migration. On internal validation, leak prediction demonstrated excellent agreement with actual locations (ICC of 0.97 for x-axis, 0.96 for y-axis), with MAE of 0.26 mm. Quadrant and hemisphere concordance of the predicted leak site falling within the correct hemisphere reached 84.1% and 97.2%, respectively. Asymmetric configurations of SRF yielded superior accuracy compared with symmetric or oval patterns. Two principal forces governed SRF distribution: centripetal vector toward fovea and gravity.

**Conclusions:**

Subretinal fluid morphology in CSCR follows predictable patterns and rules that are similar to Lincoff’s rule, being influenced by macular pooling capacity and gravity. The rules provided proof of concept for a nonangiographic approach to leak site estimation that warrants prospective validation.

**Financial Disclosure(s):**

Proprietary or commercial disclosure may be found in the Footnotes and Disclosures at the end of this article.

Central serous chorioretinopathy (CSCR) is predominantly a disease of the macular region affecting middle-aged males. It is characterized by focal disruption of the outer blood-retinal barrier and subretinal fluid (SRF) accumulation.^1^^,^^2^ Increased pressure within the dilated choroidal blood vessels causes fluid to escape through micro-rips in the retinal pigment epithelium (RPE), leading to the accumulation of SRF, which is often one of the first signs observed. Although the mechanism underlying this choroidal vessel hyperpermeability remains poorly understood, a venous overload model due to outflow obstruction has been proposed.^3^

Although the condition is often self-resolving in nature, many eyes require treatment of the leak site (detected using dye angiography) with laser or photodynamic therapy.^1^ However, there may be many limitations to performing such an invasive test, including the availability of equipment, patient compliance, and contraindications to dye injection. Several studies have explored methods of nonangiographic detection of leak site using local structural changes.^4^^,^^5^ However, these approaches can be impacted by issues in acquisition or local changes that mimic the leak site characteristics. To overcome this, we focused on a more accessible parameter—the shape and extent of SRF. The dimensions of SRF have previously been used to assess regression patterns in CSCR.^6^ In the present study, we applied these parameters to evaluate SRF accumulation patterns and to determine the likely site of primary leakage, following principles similar to those proposed by Lincoff et al^7^ for identifying the primary break in rhegmatogenous retinal detachment. Lincoff et al identified that SRF in retinal detachment tends to follow predictable patterns influenced by gravity and the location of the primary break.^7^ These principles helped surgeons pinpoint the primary break more easily, often without extensive intraoperative searching, significantly improving surgical planning for retinal detachment. We believe that similar forces may influence the distribution of SRF in CSCR and that we could develop comparable methods to identify the leak site without angiography. Our framework suggests that SRF in CSCR is primarily affected by 2 main forces: a centripetal movement driving fluid toward the fovea and gravity. The interaction between these 2 factors shapes the pattern of SRF accumulation.

## Methods

This study was designed as a hypothesis-generating, descriptive observational study. The primary objective was to characterize the morphological patterns of SRF accumulation in CSCR and to derive a conceptual framework for estimating the probable leak site from enface OCT images. Cases were gathered through a collaborative effort by members of the Macula Society to create a dataset of CSCR patients with detailed multimodal imaging. Institutional review board approval was obtained from each participating center, including a waiver of individual patient consent due to the retrospective nature of the study and the minimal risk involved; the study followed the principles outlined in the Declaration of Helsinki. A complete list of participating sites is provided in Table S1 (available at www.ophthalmologyscience.org). The inclusion criteria were: (1) age >18 years; (2) confirmed diagnosis of acute CSCR; (3) presence of a single leak on fundus fluorescein angiography (FFA) at presentation; and (4) availability of baseline OCT volume scan. Exclusion criteria included: (1) poor-quality OCT or FFA images due to media haze or artifacts, or if superior portion of SRF or the leak site was present outside the OCT scan area; (2) presence of other retinal diseases such as diabetic retinopathy, high myopia, or retinal vein occlusion that could influence the image; and (3) evidence of macular neovascularization at baseline, confirmed by OCT angiography or indocyanine green angiography, when available.

**Figure 1:**
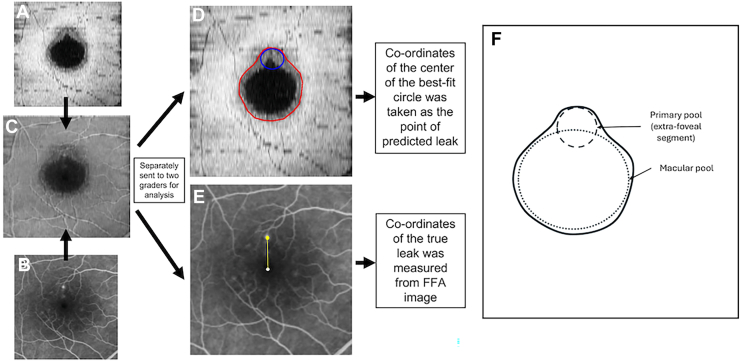
The primary image processing was done using ImageJ. **A,** The segmented en face OCT image and **(B)** an exported FFA image were cropped after superimposing using the “overlay” tool **(C)**. The boundary of SRF was also manually drawn using the “polygon” and “Draw” tool. A best-fit circle corresponding as per the rules was placed. **D,** The center of this best-fit circle (point of predicted leak) and **(E)** true leak point from FFA were noted (schematic showing the 2 segments of an asymmetric SRF collection: the primary pool and the macular pool). FFA = fundus fluorescein angiography; SRF = subretinal fluid.

Fundus fluorescein angiography images were obtained using Spectralis Heidelberg Retina Angiograph (HRA) + OCT system (Heidelberg Engineering). The earliest phase of the angiogram was used to identify the site of leakage. A volumetric OCT scan covering a 20 × 20° (6 × 6 mm) region, consisting of 49 horizontal B-scans with 25-frame averaging, was also acquired. An en face image of the outer retina was generated using a 10-pixel offset and a 5-pixel slab width, based on a previously validated algorithm for Bruch’s membrane segmentation.^8^ This provided the extent of the SRF. The primary image processing was done using ImageJ (v2.16). In eyes where the inferior portions of the SRF (scans with missing superior portion of SRF or leak site were excluded) extended beyond the 6 × 6 mm OCT scan area, infrared reflectance image (based on availability of good quality images) was superimposed using the “overlay” tool and used to delineate the missing boundary. The FFA image was cropped and resized by superimposing the FFA image over the outer retinal enface OCT image (with or without infrared reflectance image). Superimposition was performed manually by adjusting the enface OCT image opacity to 50% and aligning it using vascular landmarks, followed by marking of foveal center on both images. Registration accuracy was quantified prior to starting image analysis (Supplementary Appendix–Methods S1, available at www.ophthalmologyscience.org). The enface and FFA images were then separately analyzed. The boundary of SRF was manually drawn on the enface OCT image using the “polygon” and “Draw” tool, and the area of SRF was measured (Fig 1). The area of SRF consisted of a dark core region formed by area of greater neuro-sensory detachment, a gray outer zone formed by shallow peripheral SRF, and occasionally white zones corresponding to subretinal hyper-reflective material. All 3 zones were included in the SRF boundary determination. Patterns of SRF accumulation were subsequently documented.

**Figure 2:**
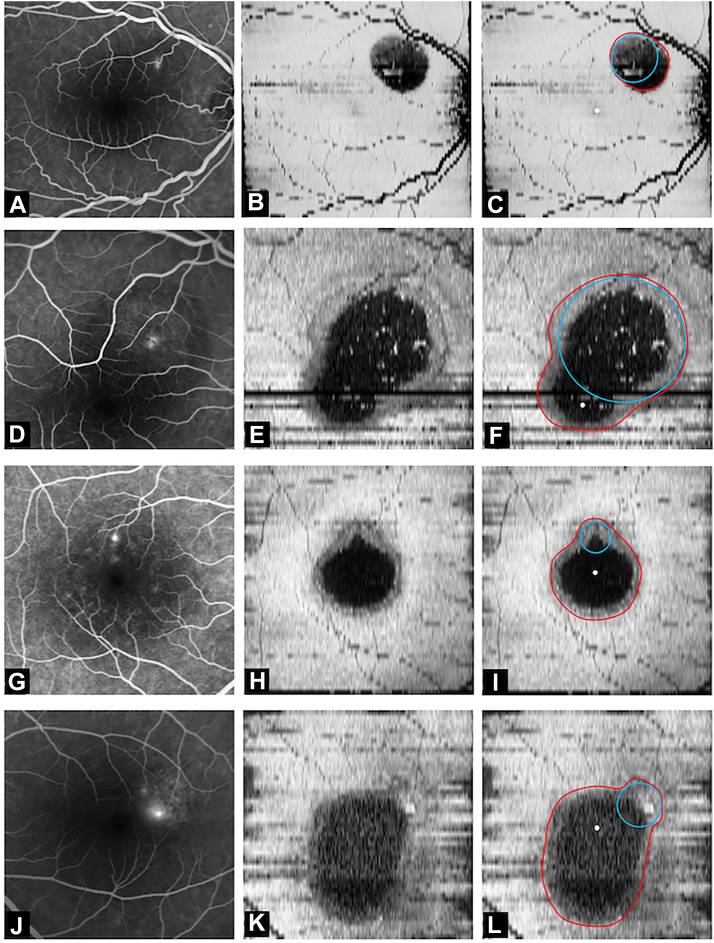
Figure showing fundus fluorescein angiography, enface OCT and marked (red line represents the border of subretinal fluid [SRF] and blue circle represents the best-fit circle) enface OCT image showing **(A–C)** pattern-1 (primary pool), **(D–F)** pattern-2 (foveal involvement), **(G–I)** pattern-3 (macular pool formation), and **(J–L)** pattern-4 (gravitating) SRF configurations.
(2) The second configuration was characterized by a pocket larger than first pattern but showing extension of the leak into the foveal region, resulting in asymmetric shape of the SRF and foveal detachment. The lesion exhibited a pear/inverted-pear morphology, characterized by a leakage point at the base (larger component) and an apex directed toward the fovea (Fig 2D, E).
(3) The third configuration had a larger detachment of the macular area. The shape of the SRF could be categorized into 2 types:
  (a) The SRF assumed a “pear-shaped” configuration. The macular detachment formed the broader portion of the pear (Fig 2G, H).
  (b) The SRF had a symmetric/near symmetrical (Fig 3A, B) or asymmetric (Fig 3C, D) oval (horizontally [more common] or vertically) configuration.

On reviewing the SRF borders, the configurations fell under 4 broad categories:

(1) The first pattern was a small circular/oval or asymmetric pocket of SRF situated away from fovea, with well-preserved vision and attached macula (Fig 2A, B).

**Figure 3:**
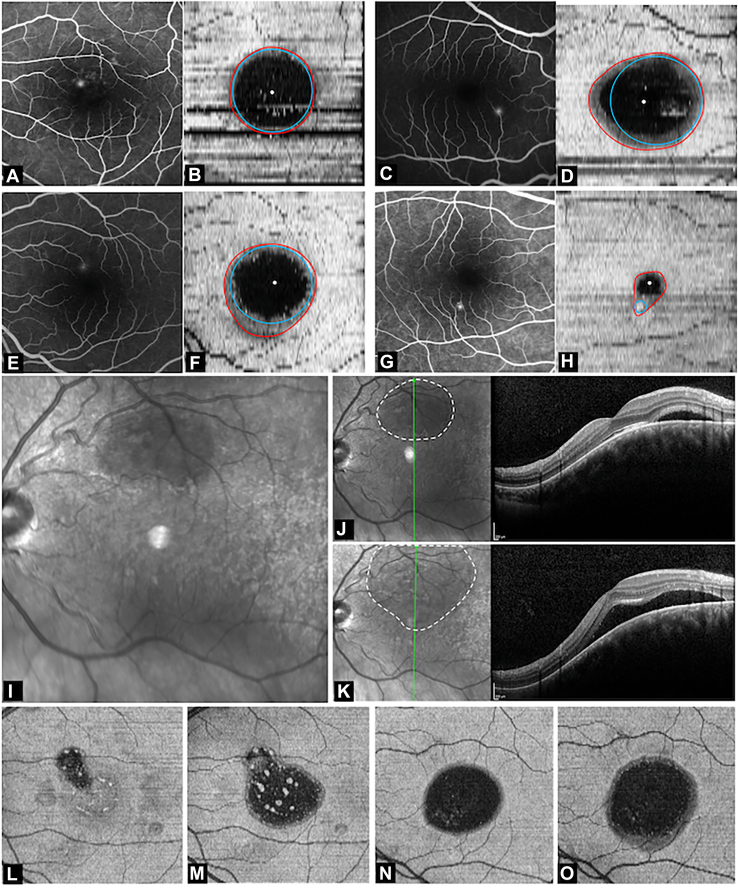
Fundus fluorescein angiography and marked (red line represents the border of subretinal fluid [SRF]; blue circle represents the best-fit circle and white dot represents center of fovea) enface OCT image in eyes with **(A, B)** near symmetrical “oval” pattern-3, **(C, D)** asymmetrical “oval” pattern-3, **(E, F)** pattern-4 with leak inside macular pool area, and **(G, H)** pattern-3 “pear” with inferior leak. **I,** Example of cases presenting with pattern-1 primary pool, **(J)** enlarging over time, and **(K)** later converting to pattern 2 due to foveal involvement of SRF; **(L)** presenting with pattern-2 at baseline and **(M)** progressing to pattern-3 in 4 months; and **(N)** presenting with pattern-3 and **(O)** progressing to pattern-4 in 3 months.
(4) The final configuration showed an asymmetric SRF collection with a longer vertical axis, extending toward or beyond the inferior macula (gravitating SRF) (Fig 2J, K). The shape had 3 parts. The superior segment/bulge had a configuration similar to the third pattern (i.e., partial pear-shaped or partial oval). The part immediately below it was either narrow or of the similar width as the superior segment. The third segment (not seen in all cases due to small scan size) was broad and constituted the inferior-most extent. The shape of SRF varied from an asymmetric vertical oval to a pear-shaped or dumbbell-like pattern.

### Determining the True and Predicted Leak Coordinates

Regardless of the patterns, majority (asymmetric) of the SRF configurations had 2 distinct segments: one around the foveal region and one extending away from it (Fig 1F). While the perifoveal segment was common to most configurations, the extrafoveal segment varied significantly between eyes. Comparison with corresponding FFA images revealed that the leak corresponded to the approximate center of the extrafoveal extension in several cases. Based on this observation, it was hypothesized that the extrafoveal segment represents the primary site of SRF accumulation around the leak and the perifoveal segment represents a secondary accumulation. To test this hypothesis, the predicted leak site was determined by placing a best-fit circle corresponding to the extrafoveal SRF segment (Fig 1) (using the “oval” tool in ImageJ) using the following rules:

(a) Pattern 1: Conforming to the top-most margin of SRF (Fig 2A–C).
(b) Pattern 2 and “pear-shaped” pattern 3: Circle was fit conforming the outer-most curvature (curvature farthest from foveal center) of the SRF border (Fig 2D–I).
(c) Oval pattern 3: Conforming to the top-most margin of SRF, in symmetrical/near-symmetrical oval SRF (Fig 3A, B). In case of asymmetric oval, the best-fit circle was fitted to the outer-most curvature (curvature farthest from foveal center) of the SRF border (Fig 3C, D).
(d) Pattern 4 (gravitating SRF): Circle was fit based on the configuration of the superior part of the SRF. If it had a single hill (single superior curve) configuration (Fig 3E, F), the best-fit circle was placed at the topmost margin of SRF (this configuration was commonly seen for perifoveal leak and SRF collections not involving fovea). In eyes where the superior region had multiple hills/pear-shaped configuration (Fig 2J–L), creating an irregular (multiple superior curves) or a broad superior hill, the best-fit circle was placed in the nonmacular hill/margin (this configuration was more common in leaks present nasal or temporal to fovea with the SRF collection involving the fovea).

It was made sure that a best-fit circle conformed to the curvature of the area where it was placed. For the purpose of this study, 2 descriptive terms were introduced to characterize the distinct zones of SRF accumulation. The extrafoveal SRF collection surrounding the leak site, located outside the macular area, was termed the “primary pool,” representing the earliest and most proximate accumulation of fluid around the leak. The SRF collection within the macular area (with or without extramacular extension), which displayed a uniform, horizontally oval or circular shape in all cases except pattern 1, was termed the “macular pool,” representing the region around the fovea prone to preferential SRF accumulation due to posterior pole curvature, with a finite capacity to hold fluid (Fig 1). Once this capacity is exceeded, gravity becomes the dominant vector, driving inferior extension of SRF. After setting the center of the fovea as reference point (x = 0, y = 0), the coordinates of the center of the best-fit circle (using “centroid” function) were recorded. The center of this best-fit circle was taken as the point of predicted leak. The SRF margin and the true leak on the FFA image were marked by one grader (G.G.), and the predicted leak (best-fit circle placement) on the enface OCT image was marked independently by a second grader (S.A.D.), with each grader masked to the other’s markings. This was done after establishing good interobserver agreement (intraclass correlation coefficient [ICC] >0.9, in a sample of 20 eyes) for both true and predicted leak marking with 2 other graders (S.A. and N.K.S.), as detailed in Supplementary Appendix-Results S1. The distance of the true and predicted leak points from the center of the fovea, per eye, was calculated as the Euclidean error as:

$${error}_{i}=\sqrt{{\left(X_{p,i}–X_{t,i}\right)}^{2}+{\left(Y_{p,i}–Y_{t,i}\right)}^{2}}$$

*X*_*p*_ = x-axis coordinates of predicted leak site; *X*_*t*_ = x-axis coordinates of true leak site; *Y*_*p*_ = y-axis coordinates of predicted leak site; *Y*_*t*_ = y-axis coordinates of true leak site.

The macular pool area was computed using a Python-based spatial overlap analysis of aligned SRF masks from pattern-4 eyes, using a 50% presence threshold to delineate the region where SRF was consistently present in at least half the contributing eyes; full details are provided in Supplementary Appendix–Methods S2.

### Statistical Analysis

All analyses were performed using R Studio (version 2025.09.1 + 401; R Foundation for Statistical Computing). Continuous variables were expressed as mean ± standard deviation, and categorical variables as counts and percentages. Normality was checked using Shapiro-Wilk test. The prediction rules were derived and internally validated on the same cohort. Because pattern-specific prediction rules were used, accuracy was reported for the entire group and separately for each SRF patterns. The accuracy of the predicted leak site was assessed using mean absolute error (MAE), median error, standard deviation, and root mean square error calculated from the Euclidean distance between the true and predicted leak coordinates across all eyes.

$$\text{MAE}=\frac{1}{n}\sum_{i=1}^{n}\left|{error}_{i}\right|\;\text{RMSE}=\sqrt{\frac{1}{n}\sum_{i–1}^{n}{{error}_{i}}^{2}}$$

Accuracy was further evaluated by determining whether the predicted and true leak sites were located in the same retinal quadrant or hemisphere (horizontal or vertical). Intraclass correlation coefficients were calculated to assess agreement between true and predicted leak coordinates, and Bland–Altman plots were generated to visualize the level of agreement. The relationship between the distance of the true leak from the fovea and various quantitative parameters (including SRF area and the best-fit circle area) was examined using Pearson correlation coefficient (r). Receiver operating characteristic curve analysis was performed to assess SRF area for discrimination of pattern 4 from pattern 3 eyes and distance of true leak from the fovea predicting symmetrical/near-symmetrical SRF configuration (oval pool). Comparisons of continuous variables between patterns were made using Kruskal–Wallis H test, followed by post hoc pairwise comparisons performed using Wilcoxon rank-sum tests with Benjamini–Hochberg correction for multiple comparisons. A *P* value <0.05 was considered statistically significant.

## Results

### Leak Characteristics

A total of 107 eyes from 106 patients (71 males and 35 females) with single leak point were analyzed. The mean age of the cohort was 45.4 ± 10.8 years. Eleven eyes (10.3%) had a smokestack pattern of leakage, and the remaining 96 eyes (89.7%) had an ink-blot pattern. A total of 76 eyes (71.1%) had a leak in the superior half of the macula and 31 (28.9%) had a leak in the inferior half. The mean distance from the center of the fovea to the true leak was 1.14 ± 0.6 mm (range: 0.13–3.59 mm). A total of 79 leaks (73.8%) were present within the 3-mm circle, 26 leaks (24.3%) were present between the 3-mm and 6-mm circle, and 2 leaks (1.9%) were present beyond the 6-mm circle.

### SRF Configurations

**Figure 4:**
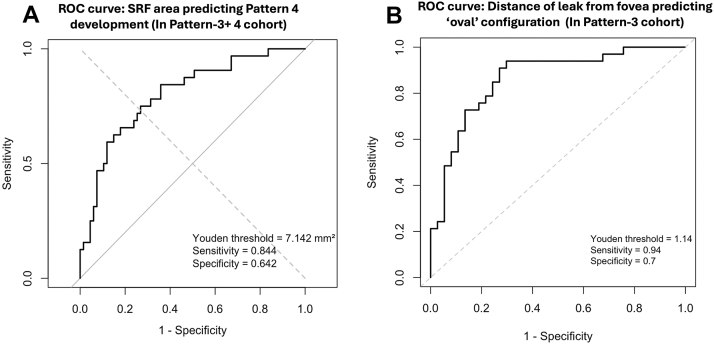
Receiver operating characteristic (ROC) curves for subretinal fluid (SRF) area predicting **(A)** Pattern-4 development and **(B)** distance of leak from fovea predicting “oval” configuration.

The first pattern of SRF was seen in 5 eyes (4.7%). The true leak point was 1.8 ± 0.5 mm (range: 1.01–2.19 mm) from the center of fovea (4 superiorly and 1 inferiorly). The mean area of SRF was 3.1 ± 2.1 mm^2^ (range: 1.47–6.38 mm^2^). The second pattern was seen in 3 eyes (2.8%). The true leak was 2.1 ± 1.3 mm (range: 0.92–3.59 mm) from the center of the fovea (all 3 leak sites superiorly). The mean area of SRF was 7.1 ± 4.9 mm^2^ (range: 1.4–10.17 mm^2^). All 3 eyes had an inverted pear-shaped configuration. The third pattern was seen in 67 eyes (62.6%), and the true leak was 1.1 ± 0.6 mm (range: 0.13–2.98 mm) from the center of the fovea (49 superiorly and 18 inferiorly). The mean area of SRF was 6.9 ± 4.9 mm^2^ (range: 1.1–20.26 mm^2^). Oval pool was noted in 33 eyes (49.3%) (17 symmetrical/near symmetrical and 16 asymmetrical) and 34 eyes (50.7%) showed a pear-shaped configuration. The fourth pattern was seen in 32 eyes (29.9%) and the true leak was 1.1 ± 0.6 mm (range: 0.33–3.36 mm) from the center of the fovea (20 superiorly and 12 inferiorly). The mean area of SRF was 16.8 ± 20.6 mm^2^ (range: 2.6–125.21 mm^2^). All eyes had a “pear”-shaped configuration (or modified pear, consisting of a vertically oval or dumbbell shape). The SRF area differed significantly between groups (*P* < 0.001). On pairwise comparison, pattern-4 had significantly higher SRF than pattern-1 and pattern-3 (*P* < 0.001 for each). Four eyes in the pattern-4 group had no macular involvement. The leak points were in the inferotemporal quadrant in all 4 eyes and were located at a mean distance of 1.5 ± 0.2 mm (range: 1.3–1.7 mm) from the center of the fovea. The receiver operating characteristic analysis of SRF area to discriminate between pattern-4 and pattern-3 eyes (from the pattern-3 and pattern-4 cohorts) showed an area under the curve of 0.8 (95% confidence interval [CI]: 0.7–0.9), indicating good discrimination. The optimal SRF area threshold based on the Youden index was 7.14 mm^2^, with a sensitivity of 84% and specificity of 64%, indicating the cut-off area beyond which pattern-4 was more common (Fig 4).

Longitudinal follow-up was seen in 73 eyes, of which 48 (65.7%) showed resolution of SRF (without any increase in SRF size) and 9 eyes (12.3%) had stable SRF during follow-up. An increase in SRF or change in pattern was seen in 16 eyes (21.9%). Of these 16 eyes, 8 eyes (50%) had increase in size of SRF, while 8 (50%) had increase in size and a change in the pattern of SRF (5 eyes had a change from pattern-3 to pattern-4 [Fig 3N, O] and 1 eye each changed from pear shape to oval shape, pattern-1 to pattern-2 and pattern-2 to pattern-3 [Fig 3L, M]).

### The Primary and the Macular Pool

**Figure 5:**
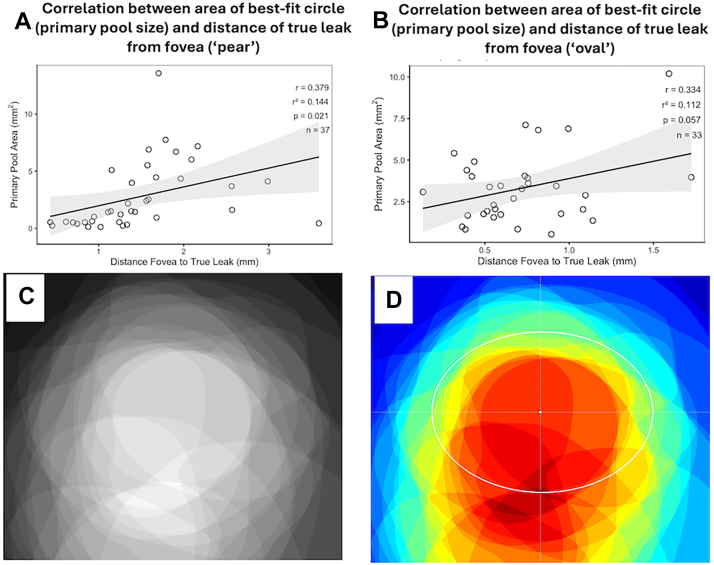
**A** and **B,** Graphs showing correlation of the area of best-fit circle (size of primary pool) with the distance of true leak from fovea, for “pear-shaped” and “oval” configurations, respectively. **C,** Grayscale overlap map showing the superimposition of aligned subretinal fluid (SRF) masks from all pattern 4 cases. All masks were spatially aligned with the foveal center as the reference point. **D,** Heat map representation of SRF spatial distribution with best-fit ellipse overlay. Color intensity indicates the frequency of SRF occurrence. The white ellipse delineates the central region of maximum SRF overlap, fitted to areas where overlap intensity exceeded 50% of maximum.

The mean area of the best-fit circle was 3.6 ± 3.2 mm^2^ (range: 0.12–13.57 mm^2^). In eyes with asymmetric pear-shaped configuration in pattern 2 and pattern 3, the size of the best-fit circle showed moderate positive correlation (r = 0.38, *P* = 0.02) with the distance of the true leak from center of fovea (Fig 5A). Similarly, there was a near-significant positive correlation seen in eyes with oval (in pattern 3) configuration (r = 0.34, *P* = 0.06) (Fig 5B). A macular pool was seen in 95 eyes (88.7%). On analysis of super-imposed SRF masks from pattern-4 cases, the median macular pool area was found to be 8.23 mm^2^, with the major and minor axes being 3.8 and 2.7 mm, respectively (Fig 5C, D).

### True vs. Predicted Leak Site

**Figure 6:**
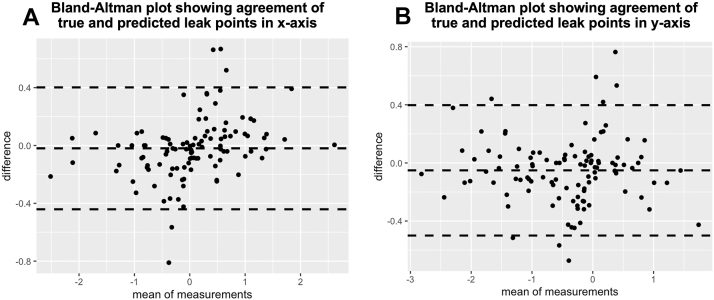
Bland-Altman plots showing agreement between true and predicted leak points in **(A)** x-axis and **(B)** y-axis.

**Figure 7:**
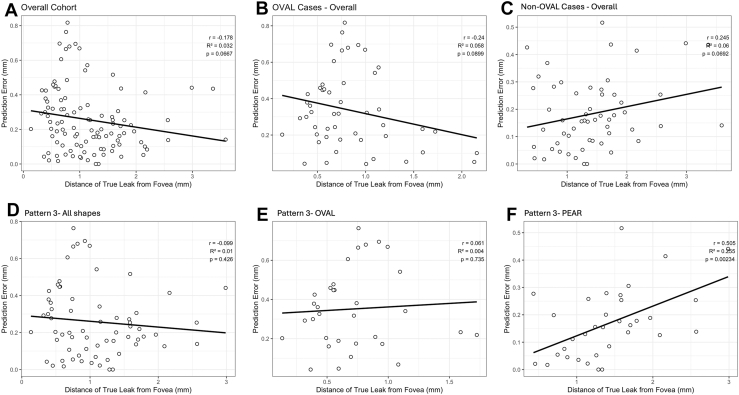
Correlations between the distance of leak site from fovea vs. the error values in different patterns of SRF. Weak, near-significant correlations were observed in the overall cohort **(A)**, all 'oval' cases **(B)**, and all 'non-oval' cases **(C)**. Meanwhile, a good correlation was observed in the asymmetric pattern-3 ('pear-shaped') SRF **(F)**. Other configurations showed poor correlations **(D, E)**. SRF = subretinal fluid.

A total of 60 eyes (56.1%) had an error of ≤0.25 mm, 86 eyes (80.4%) had an error value of ≤0.5 mm, and 107 eyes (100%) had error values ≤1 mm. Intraclass correlation coefficient was 0.97 (95% CI: 0.95–0.98, *P* < 0.001) for the x-axis and 0.96 (95% CI: 0.95–0.98, *P* < 0.001) for the y-axis. These high ICC values suggest that the predicted leak coordinates are very close to those of the true leak site. Figure 6 shows the Bland-Altman plot for the 2 axes, with bias of –0.02 mm (limits of agreement of –0.4 to 0.4) for the x-axis and –0.05 mm (limits of agreement of –0.5 to 0.4 mm) for the y-axis. In the overall cohort, the MAE, median error, standard deviation, and root mean square error were 0.26, 0.21, 0.19, and 0.32 mm, respectively. The predicted leak was in the same quadrant in 90 eyes (84.1%), same horizontal hemisphere in 97 eyes (90.6%), same vertical hemisphere in 97 eyes (90.6%), and same hemisphere (horizontal or vertical) in 104 eyes (97.2%). On subgroup analysis, eyes with nonoval (asymmetric) SRF configuration had better accuracy than those with symmetrical or near symmetrical oval leak (*P* < 0.001) (Table 1). There were differences in terms of patterns too (*P* = 0.03). Pattern 1 had significantly better accuracy than pattern 3 (*P* = 0.04) and pattern 4 (0.01). In pattern 3, symmetric/near symmetric oval pool had significantly worse accuracy compared with “pear” (*P* < 0.001) and asymmetric oval (*P* = 0.001). Numerically, high accuracy was noted in pattern 1, pattern 2, and asymmetric “pear” pattern 3 (although the number of eyes was less in pattern 1 and 2). The worst accuracy was seen for symmetric/near symmetric oval pool in pattern 3. There was a near significant negative correlation between the distance from fovea to true leak site, and error value, in the overall cohort (r = –0.2, *P* = 0.06) (Fig 7A). However, the best correlation was seen in the asymmetric pattern-3 SRF (r = 0.5, *P* = 0.002) subset, showing that leaks closer to fovea had better accuracy in this configuration of SRF (Fig 7F). Other configurations had poor correlations (Fig 7B–E). There was no difference in the MAE values between “smoke-stack” and “ink-blot” leak (*P* = 0.69) and between superior and inferior leak (*P* = 0.45).

**Table 1:** Comparison of Accuracy of Predicted Leak Site

	MAE	Median Error	SD	RMSE
Pattern 1	0.07	0.08	0.03	0.08
Pattern 2	0.18	0.16	0.04	0.18
Pattern 3	0.26	0.20	0.19	0.32
Symmetrical/near symmetrical oval pool	0.45	0.45	0.16	0.48
Asymmetrical oval pool	0.25	0.19	0.19	0.31
“Pear” shape	0.17	0.16	0.13	0.21
Pattern 4	0.29	0.25	0.19	0.34
Overall cohort	0.26	0.21	0.19	0.32
Oval pool (pattern 1+ oval pattern 3)	0.34	0.32	0.21	0.37
Nonoval pool (pattern 2+ pear-shaped pattern 3 + pattern 4)	0.18	0.16	1.13	0.22

MAE = mean absolute error; RMSE = root mean square error; SD = standard deviation.

Receiver operating characteristic curve demonstrating the cut-off for distance of true leak from fovea, closer to which a macular symmetric oval SRF was more probable (and thus the poorest accuracy), had an area under the curve of 0.86 (95% CI: 0.75–0.94), with a threshold distance of 1.14 mm (sensitivity of 0.94 and specificity of 0.7) (Fig 4B).

## Discussion

The results of this study demonstrate that SRF accumulation in CSCR follows 4 major patterns. These patterns could be used to map the most probable site of the true leak. These findings provide proof of concept that SRF morphology in CSCR follows predictable patterns, enabling estimation of the probable leak site without dye-based angiography. The study also highlighted the possibility of 2 major vector forces (the macular pool and gravity), responsible for directing the SRF into various areas of the posterior pole.

### SRF Configurations

**Figure 8:**
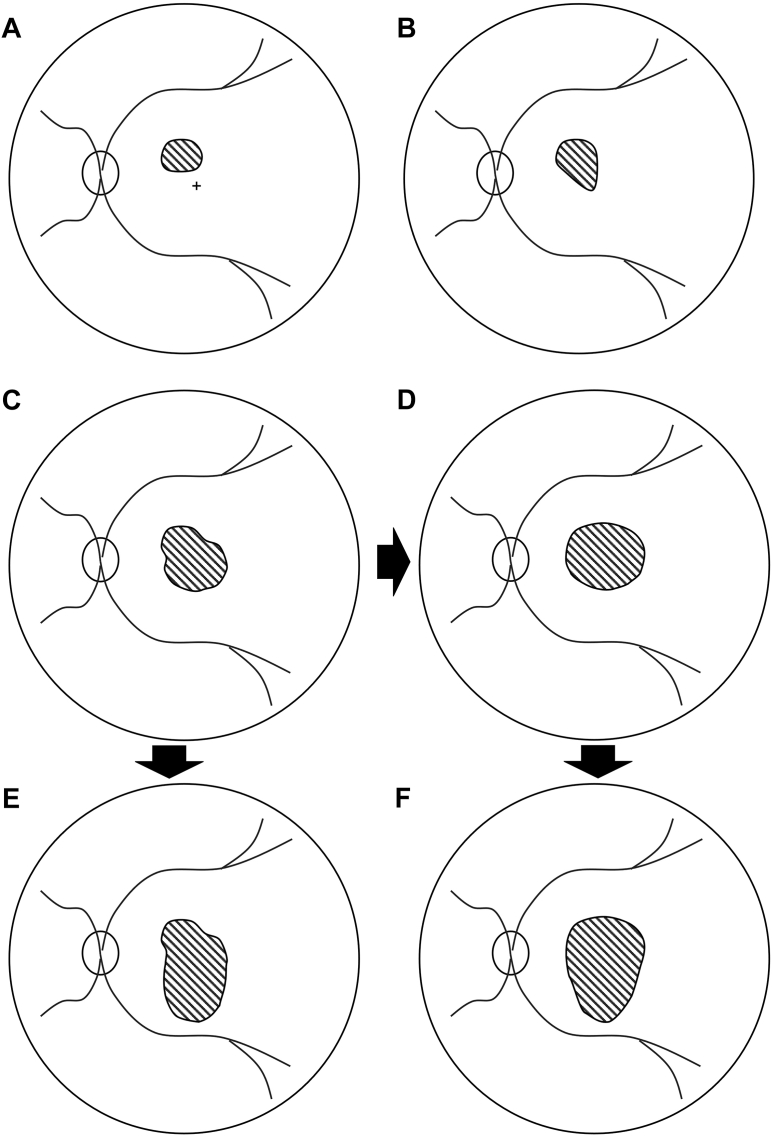
Schematic showing the hypothetical patterns of subretinal fluid (SRF) distribution, inferred from cross-sectional observations. **A,** Pattern 1: representing the primary pool configuration; **(B)** pattern 2: hypothesized foveal extension; **(C)** pattern 3: pear-shaped macular pool configuration; **(D)** pattern 3: oval configuration representing progressive macular pool expansion; **(E)** pattern 4: hypothesized inferior gravitational extension; and **(F)** pattern 4: oval macular collection with subsequent gravitational spread. Arrows represent a hypothetical progression sequence.

The patterns of SRF fell into 4 major categories—an extramacular small collection of SRF, accumulations with extensions toward the fovea, expansion of the SRF in the macular area, and the subsequent extension of SRF inferiorly. Because patients typically present to clinics only after the fovea is involved, most cases in our cohort were in pattern 3 or pattern 4. These major categories could be further reclassified based on the shape of the SRF into pear, symmetric/near-symmetric oval, or asymmetric oval shapes. These shapes may represent a sequence of SRF accumulation. Pattern 1, having a very low mean SRF area, could represent the earliest configuration of primary pooling around the leak (Fig 8A). As the SRF continues to accumulate around the site of leakage, extensions into other areas (most commonly the fovea) are seen (Fig 8B). This results in a pear (inferior leak) or inverted pear (superior leak) shape with the smaller segment at the fovea and the broader segment away from the fovea (pattern 2, characterized by foveal involvement). As SRF continues to increase further, the macular end of the SRF tends to expand concentrically to create a macular pool (secondary pool, seen in pattern 3). If the leak site remained outside the accumulated macular pool, a pear-shaped configuration (Fig 8C) is seen (broader segment at the macular area and smaller segment away from the macula). As the pool enlarges and approaches/covers the leak site, the configuration gradually transitions from an asymmetric, symmetric, or near-symmetric oval (Fig 8D). Thus, considering the fact that the SRF starts from pattern 1, the best-fit circle indirectly represented the primary pool. Interestingly, we also observed a positive correlation between the size of the primary pool (area of the best-fit circle) and the leak’s distance from the foveal center, indicating that leaks located farther from the fovea tend to enlarge more, before eventually involving the fovea. The final configuration was observed when the SRF extended inferiorly (Fig 8E, F), likely under the influence of gravity (pattern 4 characterized by the gravitation of the SRF). This inferior spread most often occurred vertically below the centroid of the pattern 2 or pattern 3 SRF collection (Fig 3E, F). This pattern had varying shapes and sizes, with many eyes having a bulbous inferior segment (forming the tertiary pool). Theoretically, early pattern-4 SRF (SRF that is beginning to extend downward) may resemble the pear-shaped pattern-3 SRF in which the leak is located inferior to the fovea (Fig 3G, H). However, the gravitational pattern-4 distribution is characterized by a larger extent of SRF and a smoother, gradually tapering transition rather than the well-defined pear-shaped contour observed in pattern 3.

### The Ocular Vectors

The direction in which a segment of SRF extended was variable. Analyzing the common patterns provided clues regarding the 2 major vectors—the macular pool and gravity. Macular pooling of SRF was seen in 89% of all cases, demonstrating the preference of SRF toward the macular area. The tendency of SRF to move toward the macular pool could be explained by the shape and curvature of RPE at the posterior pole. Previous studies have shown that the curvature of the posterior pole is greater toward the center and tends to flatten as we move to the periphery.^9^ This facilitates a greater vector force toward the center of the curve (most dependent portion in supine position), that is, the fovea. Interestingly, the macular pool was found to have a horizontally oval configuration (Fig 5D). This could have been due to the posterior pole being more curved in the vertical meridian than in the horizontal meridian,^10^ with the greater curvature area allowing for less spread of SRF than the lesser ones. Other possible uninvestigated factors could be the degree of adhesion between the photoreceptors (PRs) and the RPE, and relative position with respect to the optic disc, which might limit the expansion of SRF. We believe that most eyes can only accumulate a certain amount of SRF in the macular pool. Once this limit is reached, gravity likely becomes the primary force driving the SRF downward. However, this idea remains inferential and requires prospective longitudinal confirmation. The variability in the size of the macular pool seen in pattern-4 eyes could indicate that each eye could be harboring different capacity to hold SRF in the macular area, which could vary based on whether the posterior pole is concave (higher capacity and greater macular detachment) or dome-shaped (lesser capacity and lesser macular detachment). In our study, the receiver operating characteristic-derived SRF area threshold distinguishing pattern-3 from pattern-4 eyes was 7.14 mm^2^, and the macular pool area estimated from spatial overlap analysis of pattern-4 eyes was 8.23 mm^2^. Although numerically similar, both values were derived from the same cohort and should be interpreted as internally consistent rather than independently validated estimates. It is crucial to note that leaks closer to the fovea could tend to collect SRF in the macular pool before gravity causes it to move downward (Fig 3G, H), and leaks away from the fovea could have more effect of gravity than the macular pool, resulting in SRF moving downward, causing minimal/no macular detachment. This could be a hypothetical progression sequence, from pattern 1 through pattern 4, that represents a conceptual hypothesis regarding the natural history of SRF accumulation in CSCR rather than a validated staging system.

### Leak Site Prediction

Using the best-fit circle method within the presumed primary pool to predict the probable leak location yielded near-accurate ICC values. The high proportion of eyes having error <0.5 mm, low bias in the Bland-Altman analysis, and high ICC values indicate that the predicted leak site falls within a clinically acceptable margin of the true leak, for treatment planning. The observed MAE of 0.26 mm and hemisphere concordance of 97.2% suggest that this approach could guide treatment decisions in routine clinical practice, particularly in settings where FFA is not feasible. However, the accuracy was better in asymmetric SRF (nonoval) and dropped as the symmetry of the SRF borders improved (worst accuracy for symmetric oval configuration in pattern 3). It is acknowledged that the overall accuracy metrics combine the performance of different patterns. The differences in accuracy across these patterns partly result from the varying geometric complexity of each configuration. Asymmetric patterns, where the primary and macular pools are more clearly separate, tend to allow more accurate placement of the best-fit circle than symmetric patterns, where the pools are less distinct. Even in asymmetric oval shapes, placement of the best-fit circle in the area farthest from the foveal circle provided good accuracy. However, in symmetric shapes, the only best-fit circle possible was the one conforming to the topmost margin of SRF, resulting in poor accuracy of the predicted leak site in pattern 3. We could find that the distance closer to which eyes were more likely to present with a symmetric/near-symmetric oval configuration was 1.14 mm, which was again within the estimated macula pool dimensions. Due to the small sample sizes in patterns 1 and 2, formal sensitivity analysis for each SRF pattern was limited. Future studies with a larger number of participants would be needed to properly assess the independent effects of each rule set.

Based on the aforementioned findings, a set of rules could be identified for predicting the probable site of leakage, after making specific assumptions.

#### Assumptions

(1) The eye has a single leak, which is active.
(2) The curvature of the eye and degree of adhesion between the PRs and RPE is uniform in all directions, along the posterior pole.

#### Rules

**Figure 9:**
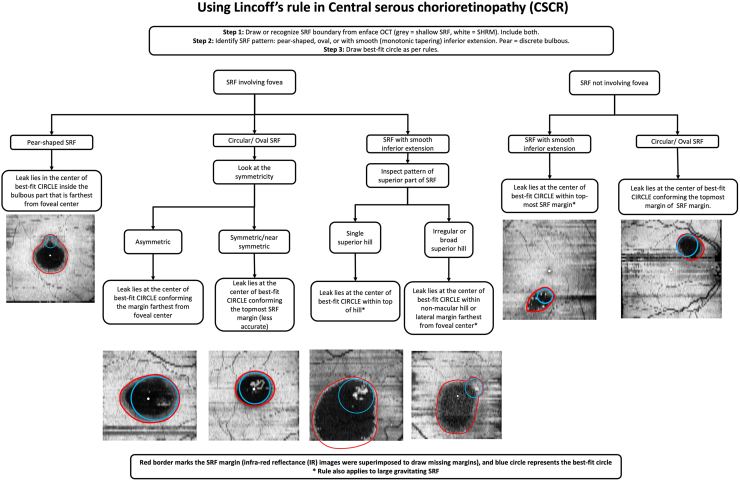
Flowchart showing the rules with representative examples. OCT = optical coherence tomography; SHRM = subretinal hyper-reflective material; SRF = subretinal fluid.
  (2) SRF has an oval configuration at macula—check if one of its margins is farther from foveal center. If the oval is:
    (a) Asymmetric—leak is located at the center of the best-fit circle conforming the margin that is farthest from foveal center.
    (b) Symmetric/near symmetric (accuracy of leak prediction is less)—leak is located at the center of the best-fit circle conforming to the topmost SRF margin.
  (3) SRF showing smooth inferior extension (characterized by smooth tapering of SRF margin below the superior segment of SRF, and not pear-shaped bulges)—observe the pattern of superior part of the SRF. If the superior part has:
    (a) A single superior hill with macular detachment and the superior border is a single smooth curve—leak is located at the center of the best-fit circle conforming within the top of the hill.
    (b) Irregular/asymmetric broad superior hill with macular detachment—the superior border has 2 curves, or a broad hill with unequal distance between the fovea and the lateral borders—leak is located at the center of the best-fit circle conforming within the nonmacular hill (or lateral margin farthest from foveal center).
B. SRF not involving fovea:
  (1) Circular or oval pocket of SRF—leak is located at the center of the best-fit circle conforming to the topmost SRF margin (accuracy will vary based on local changes in curvature).
  (2) SRF showing smooth inferior extension—leak is located at the center of the best-fit circle conforming within the top of the hill (top-most margin of the SRF).

A. SRF involving fovea:
  (1) SRF with “pear”-shaped (erect or inverted pear) configuration, with one part in the macular pool—leak is located at the center of the best-fit circle inside the bulbous region (thinner or broader region) of the pear that is farthest from foveal center (Fig 9).

#### Theoretical Exceptions

(1) Multifocal leak: Multifocal leak points may alter the configuration of the SRF. Although, theoretically, tracing each extension of the SRF (after taking the vectors into consideration) could provide the approximate location, overlapping SRF zones could make interpretation more difficult.
(2) Chronic, recurrent disease: Areas of previous detachment/SRF remnants and altered PR-RPE adhesion could alter the direction of SRF.
(3) Early pattern-4 SRF with leak within macular pool and extending vertically downward (Fig 3E, F), and pattern-3 SRF with leak site below the fovea, with SRF expanding into the macular pool (Fig 3G, H)—theoretically both can have similar configuration. However, pattern-3 leaks closer to the fovea will have smaller size. Also, leaks outside macular pool in the inferior quadrant are more likely to be influenced by gravity than the macular pool. Moreover, the smooth tapering extension of the gravitational extension of pattern 4 can be differentiated from the pear-shaped bulges of pattern-3 SRF.

The results of our study draw similarities from the principles described by Lincoff et al^7^ that guide the pattern and distribution of SRF in rhegmatogenous retinal detachment. Although we had a similar observation, the difference lay in the amount of SRF. While the major determinants of SRF configuration in rhegmatogenous retinal detachment are gravity (owing to the higher volume of SRF) and optic disc, other vectors like the centripetal vector due to posterior pole curvature play a more significant role in smaller SRF accumulations seen with CSCR.

The findings of this study have several practical implications for clinical practice. In settings with limited resources, being able to estimate the leak site noninvasively from a routinely performed OCT scan could help clinicians deliver targeted treatment without needing an invasive angiographic procedure. Also, in eyes with multifocal hyperfluorescence where it’s often difficult to identify the primary leak responsible for the SRF distribution, the pattern and direction of SRF accumulation might serve as a helpful, indirect clue to the primary leak. The clinical relevance of the reported MAE of 0.26 mm may vary by treatment modality. Although this may not be precise enough for planning a focal laser, it is likely sufficient for photodynamic therapy, as the treatment zone spans several millimeters. Additionally, tracking changes in SRF patterns over time, like a shift from an asymmetric, pear-shaped form to a more symmetric, oval shape, or the appearance of gravitational extension in the inferior part, could act as early signs of disease progression or fluid displacement. This awareness could enable intervention before significant damage to the macula occurs.

One of the biggest limitations of this study was its cross-sectional nature (although few cases with documented progression were used to verify the pattern of progression). This prevented us from quantifying the progression of SRF through the different patterns, uniformly. However, considering the often self-resolving nature of the disease and the fact that all cases in our cohort had single leak, most eyes had complete resolution of SRF, with or without treatment, without passing through the various patterns. Second, other parameters of SRF, like volumetric distribution along the SRF area, were not assessed. This could have provided better insights into the SRF migration. Third, only a small area of the posterior pole was studied. This could have prevented us from analyzing the properties of SRF originating from distant sites. Furthermore, while the SRF distribution in our study could be largely explained by 2 dominant vectors—the macular pooling force related to posterior pole curvature and the inferior gravitational vector—other biomechanical and physiological forces may also contribute. Regional variations in PR–RPE adhesion, heterogeneity in choroidal hydrostatic pressure, localized differences in RPE pump function, and structural variations in retinal curvature or rigidity could influence resistance to SRF spread. These local forces may explain some atypical or asymmetric configurations, especially in cases with near-symmetric SRF borders where our predictive accuracy was lower. Another limitation was that the prediction rules were derived and tested on the same dataset, potentially leading to circular validation and optimistic accuracy estimates. While the high ICC values and low MAE suggest strong internal consistency of the proposed framework, these metrics may not have high out-of-sample predictive accuracy. However, this MAE may be acceptable for photodynamic therapy given its larger treatment zone, but could be insufficient for focal laser photocoagulation, where precise localization within the treatment spot size is required. Future work incorporating the various structural factors and using an independent sample for validation may refine leak prediction beyond the 2-vector model used here. Additionally, these predictive rules were derived from eyes with acute CSCR showing a single active leak, and thus their relevance to chronic, recurrent, or multifocal cases remains untested. Finally, several steps in the image processing, like SRF boundary delineation, best-fit circle placement, and image registration, were performed manually, which could have introduced observer bias. Even the manual registration could have introduced an inherent error; however, residual landmark displacement in a representative subset ranged from 0.01 to 0.04 mm, suggesting a negligible contribution to the overall prediction error (Supplementary Appendix–Results S1). Nonetheless, this is the first study to assess the dynamics of migration of SRF in CSCR, thereby providing insights into the probable leak site nonangiographically.

In conclusion, 4 distinct patterns of SRF collection could be seen in CSCR, depending on the amount (area) of SRF and proposed ocular vector forces acting on them (macular pool, gravity, and local curvature changes), although the mechanistic basis of these vectors remains hypothetical and warrants prospective validation. Identifying the primary pool of SRF can help in predicting the most probable site of leakage. This information can potentially be used for targeted treatment in patients without dye angiography, which may be sufficient to guide photodynamic therapy planning, but requires prospective external validation before applying to focal laser therapy (which requires greater precision). Addressing the limitations in future studies can possibly improve the precision of leak-site prediction and help refine the rules that guide SRF migration.
