# Supplementary material for: Applying Lincoff’s Rule to Central Serous Chorioretinopathy: The Macula Society International CSCR Research Network (MICRoN) Report-15
Source: Ophthalmol Sci. 2026 Apr 17;6(6):101197. doi: 10.1016/j.xops.2026.101197 (PMC13213866; doi:10.1016/j.xops.2026.101197)
Supplement: Supplementary Appendix [file mmc1.pdf]

Supplementary methods S1. Grading and registration accuracy

Intergrader reproducibility was assessed in a sample of 20 eyes. SRF margin and true leak site marking on FFA were performed independently by two masked graders (NKS and SAD), and best-fit circle placement on enface OCT was performed independently by two masked graders (GG and SA), with discrepancies in circle placement adjudicated by a senior grader (JC). To empirically estimate the registration uncertainty inherent to this manual alignment process, a subset of [n] eyes underwent independent re-registration by two graders (GG and SAD). The displacement between corresponding vascular bifurcation points across the two registrations was measured in both x and y axes and expressed as Euclidean distance, providing a dataset-specific estimate of registration error.

Supplementary methods S2. Python-Based Spatial Overlap Analysis for Macular Pool Computation

For demonstration of the macular pool area, en face OCT images with pre-marked SRF boundaries and foveal centers of pattern-4 eyes (presuming that SRF increases in size until the macular pool limit is reached, before extending/gravitating downwards) were processed using Python (version 3.14) with OpenCV, NumPy, and PIL libraries. Binary masks were automatically generated by detecting and filling boundary contours. All SRF masks were spatially aligned using the fovea as the reference point (0,0), enabling superimposition in a common coordinate system. An overlap map was generated by summing aligned masks, creating a heatmap of SRF frequency at each location. A best-fit ellipse was fitted to the maximum overlap region by sampling radially from the fovea until overlap intensity fell below 50% of the modal overlap value, thereby delineating the core zone of most frequent and consistent SRF

occurrence across pattern-4 eyes. The ellipse was constrained to be fovea-centered and vertically symmetrical (using superior extent for both directions), while horizontal extent reflected actual nasal-temporal distribution. Major axis, minor axis, and elliptical area were also calculated. Although follow-up images of these eyes were analyzed, they were used only to confirm the progression pattern and not included in quantitative analysis because of non-uniform data capture.

Supplementary results S1: Inter-observer agreement

Intergrader agreement was excellent for the SRF area (ICC 0.97, $p<0.001$), fovea-to-true-leak distance (ICC 0.98, $p<0.001$), and fovea-to-predicted-leak distance (ICC 0.96, $p<0.001$). Based on these results, independent single readings by GG and SAD were used for the full cohort analysis, with JC adjudicating any remaining discrepancies in circle placement. The mean registration displacement (absolute) was 0.01 ± 0.01 mm (range 0.01 to 0.04 mm).
