# Supplementary material for: Applying Lincoff’s Rule to Central Serous Chorioretinopathy: The Macula Society International CSCR Research Network (MICRoN) Report-15
Source: Ophthalmol Sci. 2026 Apr 17;6(6):101197. doi: 10.1016/j.xops.2026.101197 (PMC13213866; doi:10.1016/j.xops.2026.101197)
Supplement: Table S1 [file mmc2.pdf]

Supplementary Table S1. Participating Sites

Site	Institution	Location
1	University of Pittsburgh Medical Center	Pittsburgh, PA, USA
2	Polytechnic University of Marche	Ancona, Italy
3	Retina Consultants of Texas	Houston, TX, USA
4	Inselspital, University Hospital Bern	Bern, Switzerland
5	Casey Eye Institute, Oregon Health and Science University	Portland, OR, USA
6	Asociados de Macula Vitreo y Retina de Costa Rica	San José, Costa Rica
